# Incremental diagnostic value of bloodstream pathogen ddPCR for early identification of blood culture-positive bloodstream infection

**DOI:** 10.3389/fcimb.2026.1908109

**Published:** 2026-07-29

**Authors:** Juebin Gu, Yufei Dong, Mulin Liu, Lin Lin

**Affiliations:** Department of Clinical Laboratory, The First Affiliated Hospital of Dalian Medical University, Dalian, China

**Keywords:** bacteremia, blood culture, bloodstream infection, diagnostic value, droplet digital PCR, molecular diagnostics, procalcitonin, risk stratification

## Abstract

**Background:**

Bloodstream infection (BSI) is associated with substantial morbidity and mortality. Early identification remains challenging because blood culture results are often unavailable during initial clinical decision-making. This study evaluated routinely available clinical indicators for identifying blood culture-positive BSI and investigated whether bloodstream pathogen droplet digital PCR (ddPCR) testing provides incremental diagnostic value.

**Methods:**

This retrospective study was conducted at a tertiary teaching hospital. A conventional clinical indicator cohort of 814 hospitalized patients was used to develop a prediction model for blood culture-positive BSI. An independent ddPCR cohort of 336 patients was used to evaluate the incremental value of ddPCR testing. Model discrimination, reclassification, exploratory clinical utility, and internal validation were assessed.

**Results:**

In the conventional cohort, age (OR 1.021, 95% CI 1.001–1.041, P = 0.038) and ln(PCT) (OR 1.441, 95% CI 1.203–1.727, P < 0.001) were independently associated with blood culture-positive BSI. The conventional clinical model achieved an area under the curve (AUC) of 0.696 (95% CI 0.631–0.762). In the ddPCR cohort, incorporation of ddPCR results modestly increased the AUC from 0.716 (95% CI 0.614–0.818) to 0.764 (95% CI 0.665–0.863). Although the increase was not statistically significant by the DeLong test (P = 0.152), significant improvements were observed in integrated discrimination improvement (0.041, P = 0.006) and category-free net reclassification improvement (0.786, P < 0.001). Exploratory decision curve analysis indicated higher net benefit for the combined model.

**Conclusions:**

Routinely available clinical indicators provided only modest discriminatory ability for early identification of blood culture-positive BSI. Bloodstream pathogen ddPCR testing provided additional diagnostic information with a modest and statistically non-significant increase in AUC but significant improvement in risk reclassification. ddPCR may serve as a complementary tool for early BSI risk stratification rather than a replacement for blood culture.

## 1 Introduction

Bloodstream infection (BSI) remains a major cause of morbidity and mortality among hospitalized patients and represents a substantial challenge in clinical infectious disease management (Goto and Al-Hasan, 2013; Kadri et al., 2021; Costa and Carvalho, 2022; Van Heuverswyn et al., 2023). Early recognition of BSI is critical for timely initiation of appropriate antimicrobial therapy, which is strongly associated with improved clinical outcomes (Kadri et al., 2021; Van Heuverswyn et al., 2023). However, the clinical manifestations of BSI are often nonspecific during the early stage of infection, making prompt identification difficult in routine clinical practice.

Blood culture remains the reference standard for the diagnosis of bloodstream infection (Goto and Al-Hasan, 2013; Lamy et al., 2016; Costa and Carvalho, 2022). Nevertheless, its clinical utility in early patient management is constrained by delayed pathogen identification and antimicrobial susceptibility testing, as well as by limited diagnostic yield. In clinical practice, only a proportion of patients with clinically suspected or clinically diagnosed bloodstream infection have positive bacterial or fungal blood cultures, and culture positivity may be affected by prior antimicrobial exposure, blood culture collection strategy, sampling volume, timing of collection, pathogen burden, and culture conditions. This diagnostic uncertainty creates an important clinical window period during which empirical therapeutic decisions must be made without definitive microbiological evidence. Previous studies have shown that a substantial proportion of patients with bloodstream infection receive inappropriate empiric antimicrobial therapy, which is associated with increased mortality (Kadri et al., 2021; Van Heuverswyn et al., 2023). Therefore, increasing attention has been directed toward rapidly available biomarkers and adjunctive diagnostic approaches that may facilitate earlier identification of bloodstream infection (Lamy et al., 2016; Costa and Carvalho, 2022).

Several routinely available clinical indicators, including white blood cell count (WBC), procalcitonin (PCT), and neutrophil-to-lymphocyte ratio (NLR), have been investigated for the early identification of bloodstream infection (de Jager et al., 2010; Wacker et al., 2013; Zhang and Gu, 2024). Among these biomarkers, PCT has been consistently reported as one of the most useful indicators of systemic bacterial infection and sepsis and has been widely incorporated into clinical decision-making in infectious disease management (Wacker et al., 2013; Schuetz et al., 2017; Julián-Jiménez et al., 2024). However, the diagnostic performance of conventional clinical indicators varies considerably across clinical settings, and no single biomarker has demonstrated sufficient accuracy to reliably identify all patients with blood culture-positive BSI (de Jager et al., 2010; Wacker et al., 2013). Consequently, there remains a need for diagnostic approaches that can provide additional information beyond routinely available laboratory markers.

In recent years, bloodstream pathogen droplet digital PCR (ddPCR) testing has emerged as a promising adjunctive diagnostic tool because of its substantially shorter turnaround time and ability to rapidly detect microbial nucleic acids directly from blood samples (Dunbar et al., 2022; Peri et al., 2022; Wu et al., 2022; Peng et al., 2025). Rapid molecular diagnostic assays have the potential to improve pathogen detection, facilitate earlier clinical decision-making, and support antimicrobial stewardship interventions (Dunbar et al., 2022; Peri et al., 2024). Several recent studies have demonstrated the clinical utility of multiplex PCR, ddPCR, metagenomic next-generation sequencing (mNGS), and other rapid molecular diagnostic approaches in patients with suspected bloodstream infection and sepsis (Wu et al., 2022; Liu et al., 2023; Peng et al., 2025). Nevertheless, most previous investigations have focused on pathogen detection performance and clinical impact of molecular diagnostics, whereas the incremental diagnostic value of ddPCR testing beyond routinely available clinical indicators in real-world hospitalized patients remains insufficiently characterized.

In the present study, we evaluated the diagnostic performance of routinely available clinical indicators for identifying blood culture-positive bloodstream infection in a real-world hospitalized cohort. We further investigated the incremental diagnostic value of bloodstream pathogen ddPCR testing in patients with paired ddPCR and blood culture results. The primary aim of this study was to determine whether bloodstream pathogen ddPCR testing could provide additional diagnostic information beyond routinely available clinical indicators during early diagnostic period. Accordingly, we first developed a prediction model based on routinely available clinical indicators and subsequently evaluated the incremental value of ddPCR testing in an independent ddPCR cohort.

## 2 Materials and methods

### 2.1 Study design and study population

This retrospective observational study was conducted at the First Affiliated Hospital of Dalian Medical University. Two independent analytic cohorts were established for distinct purposes: a conventional clinical indicator cohort for developing a prediction model based on routinely available clinical variables, and a ddPCR cohort for evaluating the incremental diagnostic value of bloodstream pathogen ddPCR testing beyond conventional clinical indicators.

The conventional clinical indicator cohort was derived from hospitalized patients who underwent blood culture testing between January 2024 and December 2024. Duplicate blood culture submissions during the same hospitalization were excluded. Patients younger than 18 years, non-hospitalized patients, cancelled or unreviewed reports, suspected contaminant cultures, and cases without key laboratory indicators within 72 h of blood culture sampling were excluded.

Because bloodstream pathogen ddPCR testing was introduced into routine clinical practice at our hospital in April 2025, the ddPCR cohort included hospitalized patients who underwent ddPCR testing between April 2025 and May 2026 and had paired blood culture results obtained during the same hospitalization. Repeated ddPCR tests during the same hospitalization were excluded. The ddPCR cohort was not a subset of the conventional clinical indicator cohort; the two cohorts were derived from different time periods and served distinct analytic purposes.

**Figure 1:**
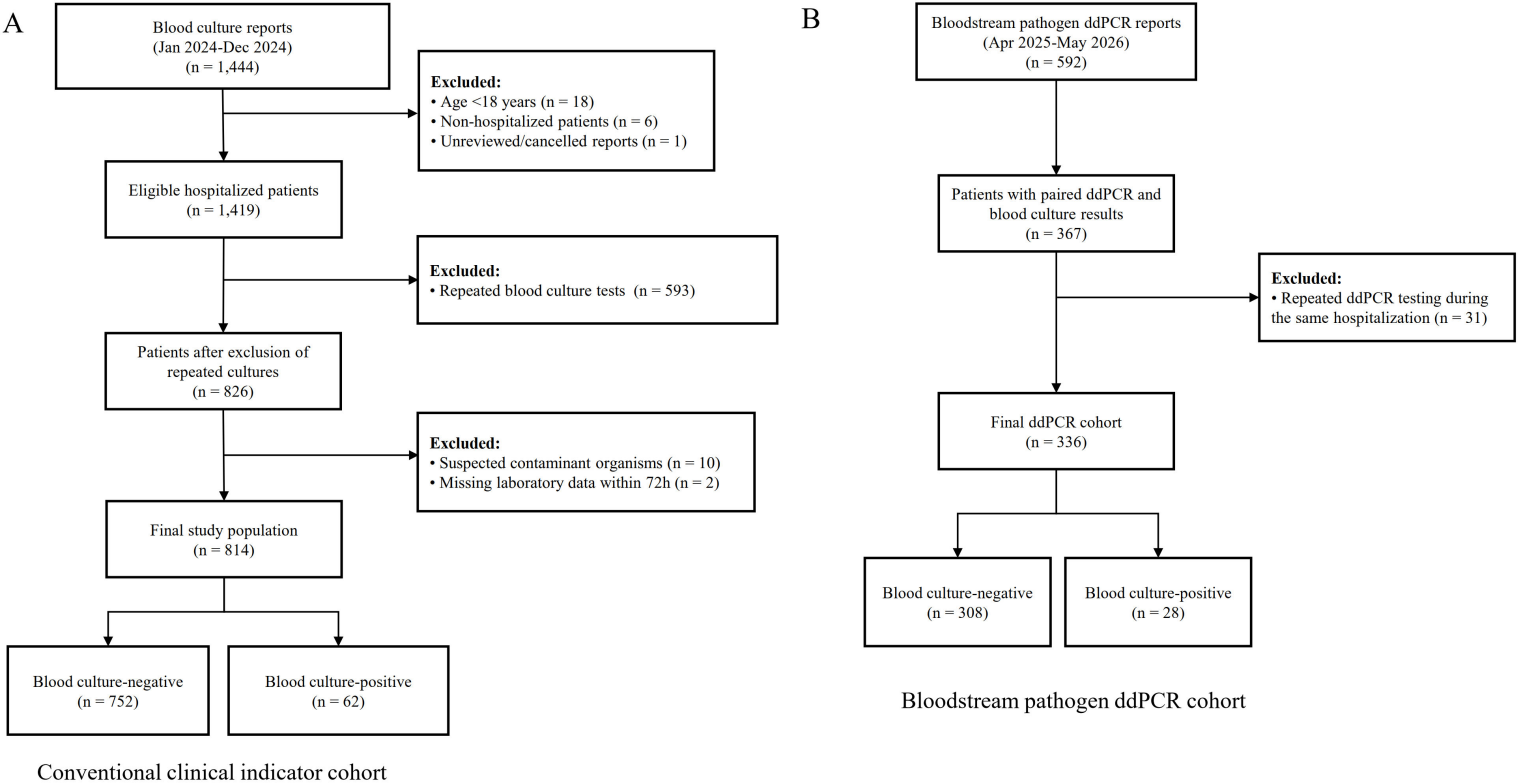
Flowcharts of patient selection. **(A)** Conventional clinical indicator cohort. **(B)** ddPCR cohort.

For both cohorts, the same hospitalization was defined as a continuous inpatient episode from admission to discharge. In the ddPCR cohort, ddPCR and blood culture results were considered paired if both tests were performed during the same hospitalization. Patient selection is shown in **Figure 1**.

This study was approved by the Institutional Ethics Committee of the First Affiliated Hospital of Dalian Medical University (Approval No. PJ-KS-KY-2026-864). Patient information was anonymized before analysis.

### 2.2 Data collection

Demographic characteristics and laboratory parameters were extracted from the electronic medical records. Continuous laboratory variables were collected from tests performed within 72 h of blood culture sampling.

For the conventional clinical indicator cohort, age, sex, white blood cell count (WBC), albumin, neutrophil-to-lymphocyte ratio (NLR), and procalcitonin (PCT) were collected.

For the ddPCR cohort, age, WBC, albumin, PCT, and bloodstream pathogen ddPCR results were collected. Neutrophil and lymphocyte percentages from the same complete blood count were additionally collected to calculate NLR for the sensitivity analysis.

Blood culture status was used as the study outcome. Patients with positive blood culture results were classified as blood culture-positive, whereas those with negative blood culture results were classified as blood culture-negative. ddPCR results were recorded as binary variables, positive or negative, according to the final laboratory reports.

### 2.3 Blood culture and ddPCR testing

Blood cultures were performed according to the routine workflow of the hospital microbiology laboratory. For adult patients, blood cultures were generally collected as one or more sets according to clinical indications, with each set consisting of one aerobic bottle and one anaerobic bottle. The recommended blood volume was 8–10 mL per bottle. Blood culture bottles were incubated using the BacT/ALERT VIRTUO blood culture system. Routine bacterial cultures were incubated for 5 days, whereas fungal cultures were incubated for 14 days when clinically indicated.

Positive blood culture bottles were processed according to standard microbiological procedures, including Gram staining, subculture, organism identification, and antimicrobial susceptibility testing. Organism identification was performed using Clin-TOF-II matrix-assisted laser desorption/ionization time-of-flight mass spectrometry, VITEK 2 Compact, or MicroScan 96 systems, as appropriate. For specific organisms, such as Salmonella species, serological agglutination testing was performed when necessary. Antimicrobial susceptibility testing was performed using VITEK 2 Compact, MicroScan 96, or the Kirby–Bauer disk diffusion method, and results were interpreted according to current laboratory standards and internal quality-control procedures. The blood culture workflow remained consistent during the study period from 2024 to 2026.

Blood culture contamination was assessed according to WS/T 503–2017 and the standard operating procedures of the hospital microbiology laboratory. Potential contamination was not determined solely by organism identity. In routine reporting, common skin commensals or other organisms suggestive of contamination were reported with interpretive comments when appropriate, and clinicians were advised to interpret these results in conjunction with the clinical context. For the present analysis, cultures were excluded as suspected contaminants only when they were classified as such in the final reviewed laboratory report. Single-set positive cultures that could not be definitively classified as contaminants were retained as reported results.

Bloodstream pathogen ddPCR testing was performed as a routine clinical diagnostic test using commercially available ddPCR-based bloodstream pathogen nucleic acid detection kits and the TC2 PCR amplification platform (Linghang Gene Technology Co., Ltd., Hangzhou, China). According to the manufacturer’s instructions and the hospital-reported assay panel, the ddPCR assay covered eight pathogen targets: *Pseudomonas aeruginosa*, *Escherichia coli*, *Klebsiella pneumoniae*, *Acinetobacter baumannii*, *Staphylococcus aureus*, *Enterococcus* spp., *Streptococcus* spp., and *Candida* spp. Positive ddPCR results were automatically determined by the assay software according to copy number-based interpretation criteria.

The decision to order ddPCR testing was made by the treating clinicians according to routine clinical practice and was independent of the present study. Laboratory personnel performed ddPCR testing in accordance with the manufacturer’s instructions and local standard operating procedures. In the present study, only previously reported ddPCR results were retrospectively collected for statistical analysis, and no additional laboratory testing was performed by the investigators. Blood culture positivity was used as the reference outcome when evaluating the diagnostic performance and incremental value of ddPCR.

### 2.4 Data preprocessing

Because PCT values showed a skewed distribution, natural logarithmic transformation was performed before analysis. Extreme WBC values > 100 × 10^9^/L were treated as missing before model development. For the NLR sensitivity analysis, NLR was calculated as the ratio of neutrophil percentage to lymphocyte percentage from the same complete blood count. Missing values in model covariates were imputed using the predefined median-based imputation procedure implemented in SPSS.

### 2.5 Development of prediction models

For the conventional clinical indicator cohort, univariable logistic regression analysis was first performed. Variables considered clinically relevant based on prior literature and biological plausibility, including age, WBC, albumin, NLR, and ln(PCT), were entered into the multivariable logistic regression model. A conventional clinical prediction model was subsequently constructed using age, WBC, albumin, NLR, and ln(PCT).

For the ddPCR cohort, two logistic regression models were constructed to evaluate the incremental diagnostic value of ddPCR beyond conventional clinical indicators. The ddPCR-cohort conventional model included age, WBC, albumin, and ln(PCT), whereas the combined model included the same variables plus ddPCR positivity. The incremental value of ddPCR was assessed by comparing these two nested models within the ddPCR cohort.

Because only 28 blood culture-positive events were available in the ddPCR cohort, the primary ddPCR-cohort models were kept parsimonious to reduce the risk of overfitting. Therefore, NLR was not included in the primary ddPCR-cohort conventional model. To address model comparability, an additional sensitivity analysis was performed by incorporating NLR into the ddPCR-cohort conventional and combined models. Predicted probabilities were generated from each model for subsequent performance evaluation.

### 2.6 Model performance evaluation

Model discrimination was assessed using receiver operating characteristic (ROC) curve analysis, and the area under the ROC curve (AUC) with 95% confidence intervals (CIs) was calculated. In the ddPCR cohort, the DeLong test was used to compare the AUCs of the ddPCR-cohort conventional model and the combined model.

The incremental diagnostic value of ddPCR was further evaluated using integrated discrimination improvement (IDI) and category-free net reclassification improvement (NRI). Decision curve analysis (DCA) was performed as an exploratory assessment of the potential clinical utility of the prediction models across a range of threshold probabilities.

### 2.7 Internal validation and calibration

Internal validation was performed using bootstrap resampling with 1,000 iterations.

Model calibration was assessed using calibration curves, calibration intercepts, calibration slopes, and Brier scores.

Bootstrap-corrected AUC values were calculated to estimate optimism-adjusted model performance.

### 2.8 Statistical analysis

Continuous variables were tested for normality before analysis. Because most continuous variables showed non-normal distributions, results were presented as median (interquartile range), and comparisons between groups were performed using the Mann–Whitney U test.

Categorical variables were presented as counts and percentages and were compared using the chi-square test.

Odds ratios (ORs) and 95% confidence intervals (CIs) were reported for logistic regression analyses.

A two-sided P value < 0.05 was considered statistically significant.

Statistical analyses were performed using IBM SPSS Statistics version 32.0 (IBM Corp., Armonk, NY, USA) and Python version 3.11. Graphs were generated using GraphPad Prism version 11 (GraphPad Software, San Diego, CA, USA).

## 3 Results

### 3.1 Study population

A total of 1,444 blood culture reports from hospitalized patients in 2024 were initially screened. After excluding patients aged <18 years (n = 18), non-hospitalized patients (n = 6), unreviewed or cancelled reports (n = 1), repeated blood culture submissions (n = 593), suspected contaminant organisms (n = 10), and cases with missing laboratory data within 72 h (n = 2), 814 patients were included in the conventional clinical indicator cohort. Among them, 62 patients were blood culture-positive and 752 were blood culture-negative (**Figure 1A**).

For the ddPCR cohort, 592 bloodstream pathogen ddPCR reports from April 2025 to May 2026 were initially identified. After matching ddPCR and blood culture reports, 367 cases remained. Repeated ddPCR testing during the same hospitalization (n = 31) was excluded, resulting in a final ddPCR cohort of 336 patients, including 28 blood culture-positive and 308 blood culture-negative cases (**Figure 1B**).

### 3.2 Baseline characteristics of the conventional clinical indicator cohort

Baseline characteristics of the conventional clinical indicator cohort are presented in **Table 1**. Compared with blood culture-negative patients, blood culture-positive patients were older (68.0 vs. 64.0 years, P = 0.017) and had significantly higher NLR values (9.88 vs. 7.00, P = 0.016). Serum ln(PCT) levels were also markedly elevated in blood culture-positive patients (0.86 vs. −0.80, P < 0.001). No significant differences were observed in sex, white blood cell count, or albumin level between the two groups (**Table 1**).

**Table 1:** Baseline characteristics of the conventional clinical indicator cohort.

Variable	Blood culture-negative	Blood culture-positive	P value
	(n = 752)	(n = 62)	
Age, years	64.0 (52.0–72.0)	68.0 (58.8–74.5)	0.017
Male sex, n (%)	437 (58.1%)	41 (66.1%)	0.218
WBC, ×10^9^/L	8.33 (5.67–11.83)	9.05 (5.86–12.70)	0.418
Albumin, g/L	35.12 (31.60–38.30)	34.65 (31.80–37.30)	0.398
NLR	7.00 (3.56–12.12)	9.88 (4.78–19.50)	0.016
ln(PCT)	-0.80 (-1.39–0.91)	0.86 (-0.60–1.11)	<0.001

Data are presented as median (interquartile range) or n (%).

P values were calculated using the Mann–Whitney U test for continuous variables and the chi-square test for categorical variables.

### 3.3 Development and performance of the conventional clinical prediction model

All clinically relevant variables were entered into the multivariable logistic regression model (**Table 2**). In multivariable analysis, age (OR 1.021, 95% CI 1.001–1.041, P = 0.038) and ln(PCT) (OR 1.441, 95% CI 1.203–1.727, P < 0.001) remained independently associated with blood culture-positive bloodstream infection, whereas NLR lost statistical significance after adjustment.

**Table 2:** Univariable and multivariable logistic regression analysis in the conventional clinical indicator cohort.

Variable	Univariable OR (95% CI)	P value	Multivariable OR (95% CI)	P value
Age, years	1.022 (1.004–1.041)	0.019	1.021 (1.001–1.041)	0.038
Male sex	1.407 (0.816–2.428)	0.220	—	—
WBC, ×10^9^/L	1.009 (0.972–1.047)	0.637	0.994 (0.945–1.044)	0.798
Albumin, g/L	0.982 (0.937–1.030)	0.460	1.015 (0.966–1.066)	0.549
NLR	1.022 (1.006–1.037)	0.007	1.007 (0.989–1.025)	0.435
ln(PCT)	1.473 (1.245–1.743)	<0.001	1.441 (1.203–1.727)	<0.001

Data are presented as odds ratio (OR) with 95% confidence interval (CI).

Univariable and multivariable logistic regression analyses were performed to identify factors associated with blood culture-positive bloodstream infection.

Variables considered clinically relevant were included in the multivariable logistic regression model.

**Figure 2:**
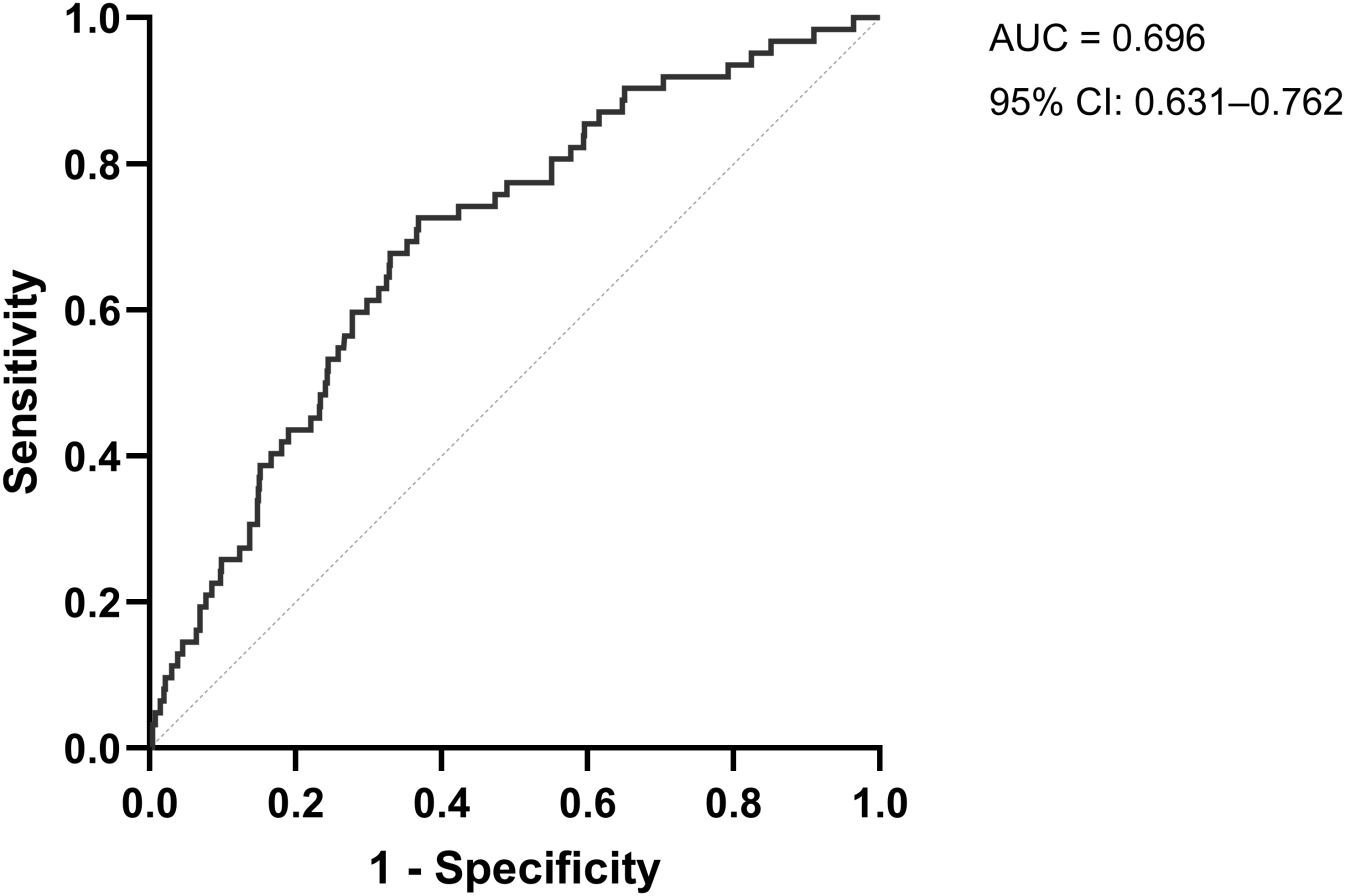
Receiver operating characteristic (ROC) curve of the conventional clinical prediction model for identifying blood culture-positive bloodstream infection in the conventional clinical indicator cohort.

A conventional clinical prediction model incorporating age, WBC, albumin, NLR, and ln(PCT) was subsequently developed. The model demonstrated modest discrimination for identifying blood culture-positive bloodstream infection, with an AUC of 0.696 (95% CI 0.631–0.762) (**Figure 2**).

### 3.4 Characteristics of the ddPCR cohort

Baseline characteristics of the ddPCR cohort are shown in **Table 3**. No significant differences were observed between blood culture-positive and blood culture-negative patients regarding age, white blood cell count, or albumin level. However, ln(PCT) remained significantly higher among blood culture-positive patients (0.28 vs. −0.97, P < 0.001).

**Table 3:** Baseline characteristics of the ddPCR cohort stratified by blood culture results.

Variable	BC-negative (n = 308)	BC-positive (n = 28)	P value
Age, years	65.0 (55.0–75.0)	58.5 (47.0–70.0)	0.382
WBC, ×10^9^/L	5.57 (3.02–10.66)	5.30 (1.40–15.70)	0.825
Albumin, g/L	33.3 (30.2–36.4)	33.3 (30.1–36.6)	0.329
ln(PCT)	-0.97 (-1.68 to -0.25)	0.28 (-1.51–2.07)	<0.001

Data are presented as median (interquartile range).

P values were calculated using the Mann–Whitney U test for continuous variables.

### 3.5 Diagnostic performance of bloodstream pathogen ddPCR

Using blood culture as the reference standard, bloodstream pathogen ddPCR demonstrated a sensitivity of 60.7%, specificity of 78.9%, positive predictive value of 20.7%, negative predictive value of 95.7%, and overall accuracy of 77.4% (**Table 4**).

**Table 4:** Diagnostic performance of bloodstream pathogen ddPCR in the ddPCR cohort.

Measure		Value		
Sensitivity		60.7%		
Specificity		78.9%		
Positive predictive value (PPV)		20.7%		
Negative predictive value (NPV)		95.7%		
Accuracy		77.4%		
Pearson χ²		21.828		
P value		<0.001		
ddPCR results versus blood culture results				
ddPCR result	Blood culture-positive (n = 28)		Blood culture-negative (n = 308)	Total
				(n = 336)
ddPCR-positive	17		65	82
ddPCR-negative	11		243	254
Total	28		308	336

PPV, positive predictive value; NPV, negative predictive value.

Blood culture was used as the reference standard.

Pearson chi-square test was used to assess the association between ddPCR and blood culture results.

A pathogen-level breakdown of discordant ddPCR and blood culture results is shown in **Supplementary Table 1**. Among the 65 ddPCR-positive/blood culture-negative cases, the most frequently detected pathogen targets were *Streptococcus* spp., *Escherichia coli*, and *Acinetobacter baumannii*. Among the 11 ddPCR-negative/blood culture-positive cases, 6 involved pathogens outside the ddPCR panel.

### 3.6 Incremental diagnostic value of ddPCR

**Figure 3:**
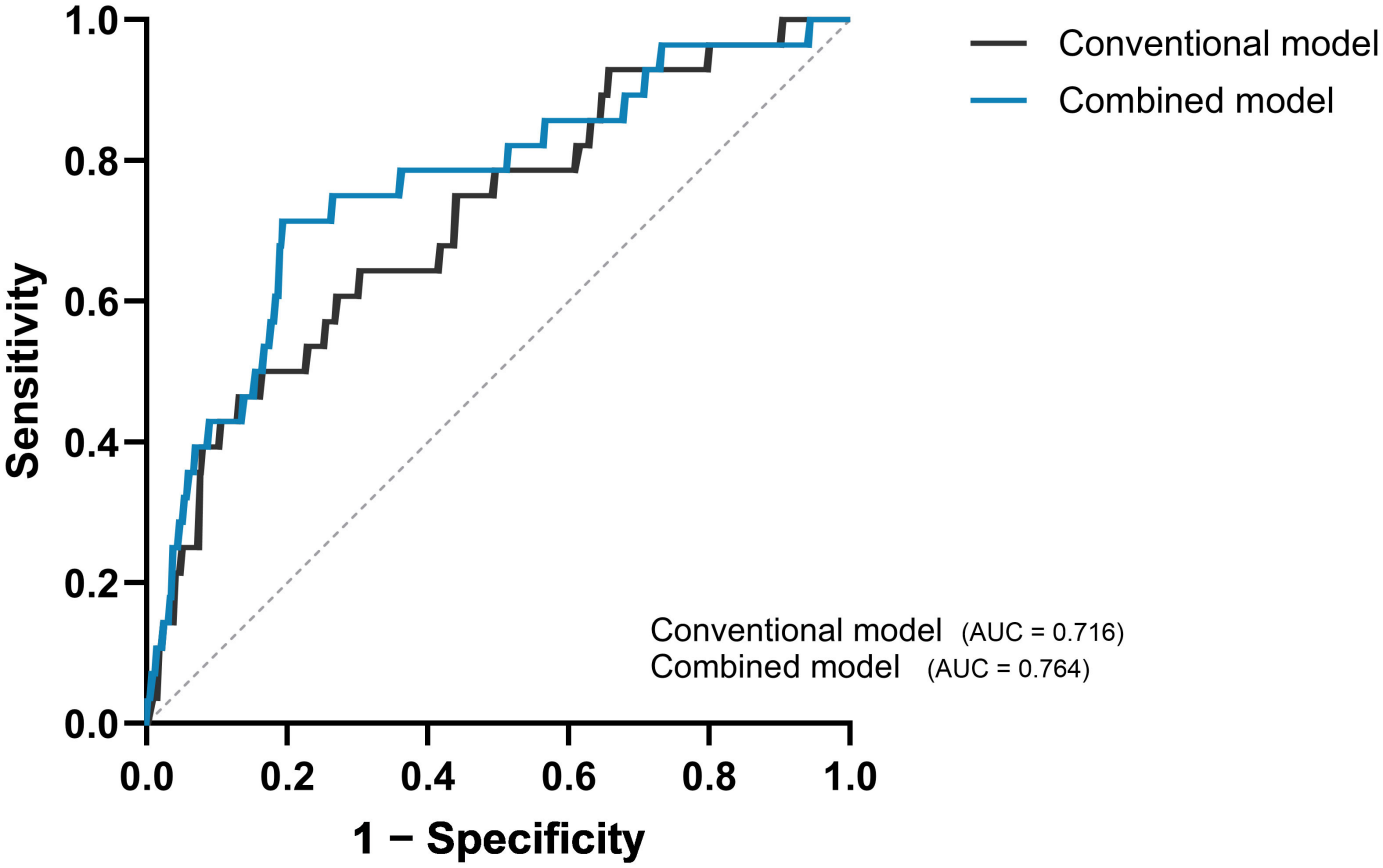
Receiver operating characteristic (ROC) curves of the conventional and combined models in the ddPCR cohort. The conventional model included age, WBC, albumin, and ln(PCT), whereas the combined model additionally incorporated bloodstream pathogen ddPCR results.

After incorporation of ddPCR results into the conventional model, the AUC modestly increased from 0.716 (95% CI 0.614–0.818) to 0.764 (95% CI 0.665–0.863) (**Figure 3**, **Table 5**), corresponding to an absolute improvement (ΔAUC) of 0.049. Although the DeLong test did not demonstrate a statistically significant difference between the two ROC curves (P = 0.152), reclassification analysis showed significant improvement after inclusion of ddPCR information, with an IDI of 0.041 (P = 0.006) and a category-free NRI of 0.786 (P < 0.001).

**Table 5:** Comparison of predictive performance between the conventional and combined models in the ddPCR cohort.

Metric	Conventional model	Combined model	Comparison
AUC (95% CI)	0.716 (0.614–0.818)	0.764 (0.665–0.863)	ΔAUC = 0.049
DeLong test (P value)	—	—	0.152
IDI	—	—	0.041 (P = 0.006)
category-free NRI	—	—	0.786 (P < 0.001)

AUC, area under the receiver operating characteristic curve;

IDI, integrated discrimination improvement;

NRI, category-free net reclassification improvement.

The conventional model included age, WBC, albumin, and ln(PCT).

The combined model further incorporated bloodstream pathogen ddPCR results.

In a sensitivity analysis additionally including NLR in the ddPCR-cohort conventional model, ddPCR remained independently associated with blood culture positivity (OR 4.108, 95% CI 1.704–9.901, P = 0.002), and the AUC increased from 0.731 for the conventional model including NLR to 0.779 after adding ddPCR (**Supplementary Table 2**). Complete multivariable logistic regression results for the ddPCR-cohort conventional and combined models are provided in **Supplementary Table 3**.

### 3.7 Decision curve analysis

**Figure 4:**
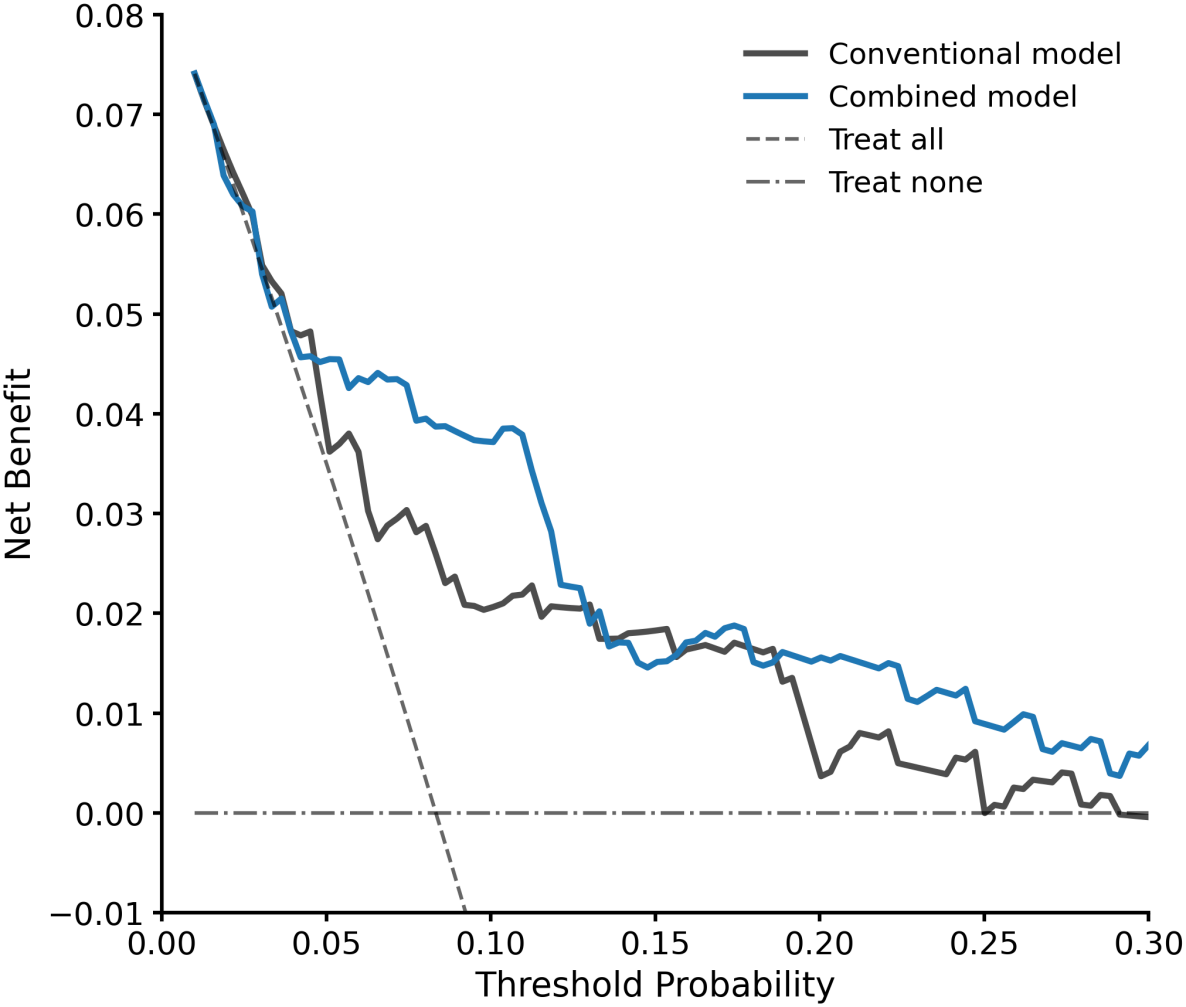
Decision curve analysis comparing the net benefit of the conventional and combined models in the ddPCR cohort.

Decision curve analysis was performed as an exploratory assessment of potential clinical utility. The combined model showed higher net benefit than the conventional model across threshold probabilities of approximately 0.05 to 0.30 (**Figure 4**). This threshold range may correspond to clinical scenarios in which a predicted 5%–30% probability of blood culture-positive BSI would prompt closer monitoring, repeat microbiological evaluation, or earlier optimization of empirical antimicrobial therapy. However, because a minimum clinically important difference in net benefit was not prespecified, these findings should be interpreted as exploratory rather than definitive evidence of clinically meaningful benefit.

### 3.8 Internal validation and calibration

**Figure 5:**
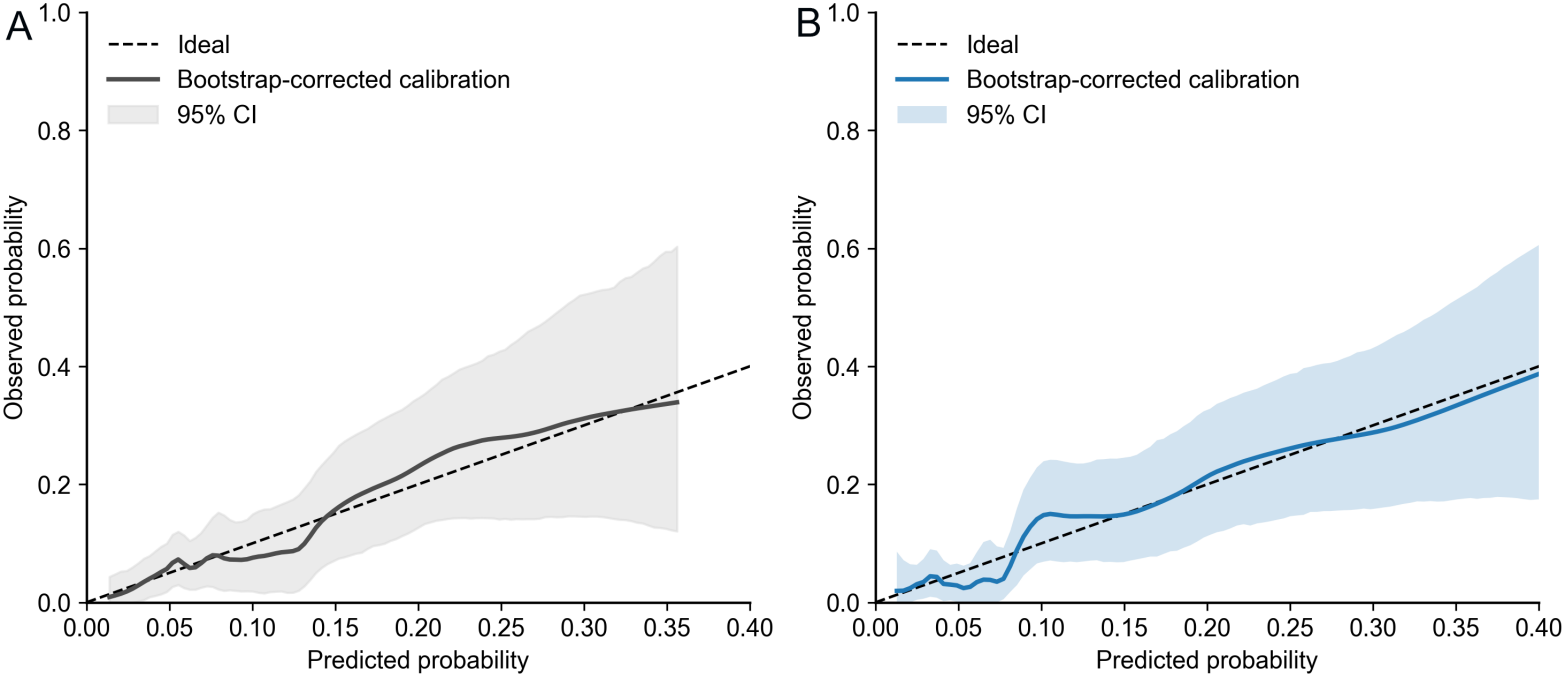
Calibration curves of the conventional and combined models after bootstrap internal validation in the ddPCR cohort. **(A)** Conventional model. **(B)** Combined model.

Bootstrap internal validation suggested acceptable discrimination and calibration. The bootstrap-corrected AUC was 0.717 for the conventional model and 0.764 for the combined model. Calibration intercepts were close to zero (0.002 and 0.001, respectively), calibration slopes were approximately one (1.001 and 1.000, respectively), and Brier scores remained low (0.071 and 0.068, respectively), indicating acceptable agreement between predicted and observed probabilities (**Figure 5**; **Table 6**).

**Table 6:** Internal validation and calibration performance of the conventional and combined models in the ddPCR cohort.

Metric	Conventional model	Combined model	Comparison
Bootstrap-corrected AUC	0.717	0.764	—
Brier score	0.071	0.068	—
Calibration intercept	0.002	0.001	—
Calibration slope	1.001	1.000	—

Bootstrap-corrected AUC, optimism-corrected area under the receiver operating characteristic curve.

Calibration intercepts close to 0 and calibration slopes close to 1 indicate acceptable agreement between predicted and observed probabilities.

## 4 Discussion

In the present study, we evaluated the diagnostic performance of routinely available clinical indicators for identifying blood culture-positive BSI and further investigated the incremental diagnostic value of bloodstream pathogen ddPCR testing. Using a conventional clinical indicator cohort, we found that age and ln(PCT) were independently associated with blood culture-positive BSI, whereas the resulting prediction model demonstrated only modest discriminatory ability. In an independent ddPCR cohort, incorporation of ddPCR results improved risk reclassification and showed higher net benefit in exploratory decision curve analysis, while modestly increasing model discrimination. Although the increase in AUC did not reach statistical significance according to the DeLong test, significant improvements were observed in both IDI and category-free NRI. These findings suggest that ddPCR testing may provide additional information for individual risk classification despite only modest changes in overall discrimination (Pencina et al., 2008). This discrepancy highlights the importance of evaluating incremental diagnostic value using multiple complementary metrics, as changes in discrimination alone may underestimate improvements in individual risk classification (Pencina et al., 2008; Kerr et al., 2014).

Blood culture remains the cornerstone of bloodstream infection diagnosis; however, its clinical utility during the initial phase of patient management is limited by the time required for pathogen identification and antimicrobial susceptibility testing, and its diagnostic yield is affected by blood culture collection strategy and sampling volume (Lamy et al., 2016; Dunbar et al., 2022). During this diagnostic window, clinicians must frequently initiate empirical antimicrobial therapy without definitive microbiological evidence. Consequently, there has been increasing interest in diagnostic approaches that can provide earlier information regarding the likelihood of bloodstream infection. In our study, ln(PCT) was the strongest laboratory predictor of blood culture positivity. In the conventional cohort, age and ln(PCT) remained independently associated with blood culture-positive BSI. These findings are consistent with previous evidence indicating that PCT reflects systemic bacterial inflammatory responses and may be useful for identifying severe bacterial infections and sepsis (Wacker et al., 2013). Nevertheless, the overall performance of the conventional clinical model remained limited, with an AUC of 0.696. This finding highlights the inherent limitations of routinely available clinical indicators and suggests that additional diagnostic information may be required to improve early identification of BSI.

The limited discriminatory performance of conventional indicators is biologically plausible. Bloodstream infection is a heterogeneous syndrome influenced by pathogen characteristics, infection source, host immune status, underlying comorbidities, and prior antimicrobial exposure. Common inflammatory markers such as WBC count and NLR may reflect systemic inflammatory responses but are not specific to bloodstream infection itself. Consistent with this concept, NLR was associated with blood culture positivity in univariable analysis but lost significance after multivariable adjustment, while WBC count and albumin did not independently predict the outcome. These observations suggest that conventional laboratory parameters may contribute to early risk assessment but are unlikely to provide sufficient discrimination as stand-alone tools for identifying blood culture-positive BSI.

A major finding of the present study is that the bloodstream pathogen ddPCR assay provided incremental diagnostic value beyond conventional clinical indicators. Using blood culture as the reference standard, ddPCR demonstrated a sensitivity of 60.7%, specificity of 78.9%, and a negative predictive value of 95.7%. The relatively low positive predictive value was likely influenced by the low prevalence of blood culture-positive cases (8.3%) in the ddPCR cohort, which was lower than that reported in many studies conducted in highly selected sepsis populations. Although the positive predictive value was relatively low, the high negative predictive value suggests that a negative ddPCR result may help reduce the likelihood of blood culture-positive BSI in selected clinical scenarios. However, it should not be used alone to exclude BSI. Similar diagnostic performance profiles, often characterized by moderate sensitivity and relatively high specificity, have been reported in previous studies evaluating multiplex PCR, ddPCR, mNGS, and other rapid molecular diagnostic technologies for suspected bloodstream infection (Dark et al., 2015; Wu et al., 2022; Liu et al., 2023; Peng et al., 2025).

In the present study, ddPCR-positive/blood culture-negative results were observed in 65 cases. These results should not be interpreted simply as false-positive ddPCR findings, because blood culture is an imperfect reference standard for molecular assays. Possible explanations include true bloodstream infection missed by culture, suppression of viable organisms by prior antimicrobial exposure, contamination, colonization, or detection of DNA from nonviable organisms. However, comprehensive case-level clinical adjudication was not available in this retrospective study, and we therefore did not attempt to classify individual discordant cases into these categories. Instead, we provided a pathogen-level breakdown of discordant ddPCR and blood culture results in **Supplementary Table 1**. In addition, because the ddPCR assay was a targeted panel covering eight predefined pathogen targets, blood culture-positive infections caused by organisms outside the panel could not be detected by ddPCR. This limited pathogen coverage likely contributed to some ddPCR-negative/blood culture-positive discrepancies, as 6 of the 11 ddPCR-negative/blood culture-positive cases involved organisms outside the ddPCR panel. Therefore, molecular diagnostic results should be interpreted in conjunction with clinical findings, conventional laboratory markers, and the predefined pathogen targets covered by the assay rather than in isolation (Dark et al., 2015; Dunbar et al., 2022).

The incremental value of ddPCR was further supported by model comparison analyses. After incorporation of ddPCR results, model discrimination improved from an AUC of 0.716 to 0.764. Although this increase was not statistically significant according to the DeLong test, both IDI and category-free NRI demonstrated significant improvement. However, because category-free NRI may be sensitive to small sample size and event imbalance, these reclassification findings should be interpreted as supportive rather than definitive evidence of clinical utility. This observation is important because AUC may be relatively insensitive to incremental improvements when baseline discrimination is already moderate or when the number of outcome events is limited, particularly in studies with relatively small numbers of positive cases (Pencina et al., 2008; Kerr et al., 2014). In contrast, IDI and category-free NRI provide complementary information by evaluating improvements in risk separation and patient-level reclassification (Pencina et al., 2008; Kerr et al., 2014). Therefore, our findings suggest that ddPCR may provide additional information for individualized risk stratification even when changes in overall discrimination appear modest.

Our findings are broadly consistent with recent studies emphasizing the clinical value of rapid molecular diagnostics for bloodstream infection management. Previous investigations have demonstrated that rapid molecular diagnostic technologies may shorten the time to pathogen identification and facilitate earlier antimicrobial decision-making, particularly when integrated with antimicrobial stewardship programs (Dunbar et al., 2022; Peri et al., 2024; Dunbar et al., 2025; Wolk et al., 2025). However, most published studies have primarily focused on analytical performance or pathogen detection. In contrast, the present study evaluated ddPCR within a clinical prediction framework and demonstrated significant improvements in risk reclassification, together with higher net benefit in exploratory decision curve analysis after incorporation of molecular test results. These findings suggest that ddPCR may provide complementary diagnostic information beyond pathogen detection alone when combined with routinely available clinical indicators during the early diagnostic period. Decision curve analysis provided exploratory support for the potential clinical utility of ddPCR incorporation. The combined model showed higher net benefit than the conventional model across threshold probabilities of approximately 0.05 to 0.30 (Vickers and Elkin, 2006; Vickers et al., 2019). This threshold range was considered clinically plausible because patients with a predicted 5%–30% probability of blood culture-positive BSI may be candidates for closer monitoring, repeat microbiological evaluation, or earlier optimization of empirical antimicrobial therapy while awaiting definitive blood culture results. However, this threshold range was not linked to a prespecified clinical decision protocol, and no minimum clinically important difference in net benefit was defined. Therefore, the DCA findings should be interpreted as exploratory and hypothesis-generating rather than definitive evidence of clinically meaningful benefit. Importantly, this approach should be regarded as a complementary risk stratification strategy rather than a replacement for blood culture, which remains essential for definitive pathogen identification and antimicrobial susceptibility testing.

Several limitations should be acknowledged. First, this was a single-center retrospective study, which may limit the generalizability of the findings. Second, the number of blood culture-positive cases in the ddPCR cohort was relatively small (n = 28), potentially limiting statistical power and contributing to relatively wide confidence intervals for some estimates. The limited number of outcome events may also have reduced the ability of the DeLong test to detect statistically significant differences in AUC between models. Third, blood culture was used as the reference standard, although it is an imperfect reference standard for evaluating molecular assays. Some ddPCR-positive/blood culture-negative results may represent true infections missed by culture, whereas others may reflect contamination, colonization, or detection of DNA from nonviable organisms. Because comprehensive case-level adjudication, including prior antimicrobial exposure, infection source, repeated cultures, inflammatory markers, clinical diagnosis, and outcomes, was not available for all discordant cases, the diagnostic performance estimates, particularly the positive predictive value, should be interpreted relative to blood culture rather than as definitive classification of true infection status. Fourth, because ddPCR testing was performed at the discretion of treating clinicians, selection bias cannot be excluded. Although the incremental value of ddPCR was assessed by comparing nested models within the same ddPCR cohort, patients selected for ddPCR testing may have differed from those not tested with respect to disease severity, prior antimicrobial exposure, suspected pathogen spectrum, or diagnostic uncertainty. Therefore, the findings from the ddPCR cohort may not be directly generalizable to all blood culture-tested patients, and estimates of diagnostic performance and incremental model value may differ in other clinical populations. Fifth, although bootstrap validation suggested acceptable discrimination and calibration, external validation in independent multicenter cohorts remains necessary before clinical implementation. Finally, the ddPCR assay evaluated in this study covered only eight predefined pathogen targets (*Pseudomonas aeruginosa, Escherichia coli, Klebsiella pneumoniae, Acinetobacter baumannii, Staphylococcus aureus*, *Enterococcus* spp., *Streptococcus* spp., and *Candida* spp.), and therefore may not detect bloodstream infections caused by organisms outside the assay panel. The relatively restricted pathogen coverage may also have contributed to false-negative ddPCR results, as 6 of the 11 ddPCR-negative/blood culture-positive cases involved organisms outside the assay panel, and may have limited the overall diagnostic performance of the assay. Consequently, the reported diagnostic performance should be interpreted within the context of the predefined pathogen targets covered by the assay.

In conclusion, routinely available clinical indicators, particularly age and ln(PCT), provided diagnostic information for the early identification of blood culture-positive bloodstream infection. Bloodstream pathogen ddPCR testing provided additional diagnostic information beyond conventional laboratory markers, with a modest and statistically non-significant increase in model discrimination but improved risk reclassification and higher net benefit in exploratory decision curve analysis when incorporated into a clinical prediction model. These findings suggest that bloodstream pathogen ddPCR may serve as a complementary tool for early BSI risk stratification while awaiting conventional blood culture results and highlight the potential role of rapid molecular diagnostics in supporting timely clinical decision-making.

## Data Availability

The raw data supporting the conclusions of this article will be made available by the authors, without undue reservation.
