# Supplementary material for: Incremental diagnostic value of bloodstream pathogen ddPCR for early identification of blood culture-positive bloodstream infection
Source: Front Cell Infect Microbiol. 2026 Jul 29;16:1908109. doi: 10.3389/fcimb.2026.1908109 (PMC13462041; doi:10.3389/fcimb.2026.1908109)
Supplement: Supplementary file 1 [file Table1.docx]

**Supplementary Table 1. Pathogen-level breakdown of discordant ddPCR and blood culture results**

**A. ddPCR-positive / blood culture-negative cases (n = 65)**

| **ddPCR-detected pathogen target** | **No. of detected targets** |
| --- | --- |
| *Streptococcus* spp. | 20 |
| *Escherichia coli* | 15 |
| *Acinetobacter baumannii* | 11 |
| *Enterococcus* spp. | 10 |
| *Klebsiella pneumoniae* | 9 |
| *Pseudomonas aeruginosa* | 6 |
| *Candida* spp*.* | 5 |
| *Staphylococcus aureus* | 4 |

**B. ddPCR-negative / blood culture-positive cases (n = 11)**

| **Blood culture pathogen** | **n** | **Included in ddPCR panel** |
| --- | --- | --- |
| *Escherichia coli* | 2 | Yes |
| *Klebsiella pneumoniae* | 1 | Yes |
| *Enterococcus faecium* | 1 | Yes |
| *Streptococcus sanguinis* | 1 | Yes |
| *Staphylococcus epidermidis* | 1 | No |
| *Enterobacter cloacae* | 1 | No |
| *Staphylococcus haemolyticus* | 1 | No |
| *Cryptococcus neoformans* | 1 | No |
| *Citrobacter freundii* | 1 | No |
| *Salmonella* Muenster | 1 | No |
| **Total** | **11** | Not applicable |

ddPCR, droplet digital polymerase chain reaction. Multiple ddPCR-detected pathogen targets in the same patient were counted separately.

The ddPCR panel coverage was determined according to the manufacturer’s target list.

Discordant cases were summarized at the pathogen level. Comprehensive case-level clinical adjudication was not performed.

**Supplementary Table 2. Sensitivity analysis including NLR in the ddPCR cohort**

| **Variable** | | **Conventional + NLR OR** | **95% CI** | **P value** | **Conventional + NLR + ddPCR OR** | **95% CI** | **P value** |
| --- | --- | --- | --- | --- | --- | --- | --- |
| **Age** | 0.990 | | 0.965–1.015 | 0.422 | 0.986 | 0.961–1.011 | 0.267 |
| **WBC** | 0.970 | | 0.915–1.029 | 0.317 | 0.968 | 0.911–1.029 | 0.301 |
| **NLR** | 1.025 | | 0.984–1.067 | 0.233 | 1.024 | 0.985–1.065 | 0.232 |
| **ln(PCT)** | 1.586 | | 1.245–2.021 | <0.001 | 1.414 | 1.104–1.812 | 0.006 |
| **Albumin** | 1.001 | | 0.921–1.088 | 0.982 | 1.012 | 0.932–1.100 | 0.771 |
| **ddPCR positivity** | — | | — | — | 4.108 | 1.704–9.901 | 0.002 |

**Model discrimination**

| **Model** | **AUC** | **SE** | **95% CI** |
| --- | --- | --- | --- |
| **Conventional + NLR** | 0.731 | 0.052 | 0.628–0.833 |
| **Conventional + NLR + ddPCR** | 0.779 | 0.049 | 0.683–0.874 |

NLR, neutrophil-to-lymphocyte ratio; WBC, white blood cell count; PCT, procalcitonin; ddPCR, droplet digital polymerase chain reaction; OR, odds ratio; CI, confidence interval; AUC, area under the receiver operating characteristic curve; SE, standard error. This sensitivity analysis was performed in the ddPCR cohort by additionally incorporating NLR into the conventional model. Missing covariate values were imputed using the same approach as in the primary analysis.

**Supplementary Table 3. Complete multivariable logistic regression results in the ddPCR cohort**

| **Variable** | **Conventional model OR** | **95% CI** | **P value** | **Combined model OR** | **95% CI** | **P value** |
| --- | --- | --- | --- | --- | --- | --- |
| **Age** | 0.990 | 0.966–1.015 | 0.416 | 0.986 | 0.962–1.011 | 0.272 |
| **WBC** | 0.981 | 0.931–1.033 | 0.457 | 0.981 | 0.931–1.034 | 0.475 |
| **Albumin** | 0.996 | 0.917–1.082 | 0.930 | 1.006 | 0.927–1.092 | 0.888 |
| **ln(PCT)** | 1.619 | 1.275–2.057 | <0.001 | 1.429 | 1.113–1.835 | 0.005 |
| **ddPCR positivity** | — | — | — | 4.078 | 1.692–9.828 | 0.002 |

WBC, white blood cell count; PCT, procalcitonin; ddPCR, droplet digital polymerase chain reaction; OR, odds ratio; CI, confidence interval.

The conventional model included age, WBC, albumin, and ln(PCT). The combined model additionally incorporated ddPCR positivity.
